# Recent Advances in the Discovery of Novel Drugs on Natural Molecules

**DOI:** 10.3390/biomedicines12061254

**Published:** 2024-06-05

**Authors:** Laura Quintieri, Leonardo Caputo, Orazio Nicolotti

**Affiliations:** 1 Institute of Sciences of Food Production, National Research Council (CNR), Via G. Amendola, 122/O, 70126 Bari, Italy; leonardo.caputo@ispa.cnr.it; 2 Dipartimento di Farmacia—Scienze del Farmaco, Università degli Studi di Bari “Aldo Moro”, Via E. Orabona, 4, 70125 Bari, Italy; orazio.nicolotti@uniba.it

Natural products (NPs) are always a promising source of novel drugs for tackling unsolved diseases [1,2,3]. Natural molecules have addressed the rational design of many synthetic small-molecule drugs [4,5,6], despite their chemotypes having higher structural complexity, heavier molecular weights (often > 500), more sp^3^ carbon atoms which amplify tridimensionality and stereocenters, more oxygen atoms, fewer nitrogen and halogen atoms, more H-bond acceptors and donors, marked hydrophilicity, and greater molecular rigidity [7]. Natural molecules are likely still inspiring drug discovery due to their incredible scaffold diversity, making them extremely unique and selective in the chemistry field [8]. Thus, developing new natural bioactive compounds and repurposing approved natural drugs are hot topics in drug discovery and medicinal chemistry [9]. Since most compounds exhibit synergistic effects [10] or share multiple targets [11,12], traditional approaches to finding potential drug candidates, such as bioassay-guided fractionation, can reduce their therapeutic efficacy. As a result, new techniques are required to produce drugs with high and multi-target activity as well as improved bioavailability [13,14,15]. For example, molecular biological techniques can increase the availability of novel compounds produced by bacteria or yeasts [16,17], and virtual screening approaches can generate screening libraries of natural compounds resembling drug-like compounds [18,19,20,21] or predict their biological activity and related molecular targets with chemical accuracy [22,23]. Moreover, advances in metabolomics have also allowed us to identify active compounds from natural product mixtures as well as to reveal synergistic effects in complex mixtures [24,25].

We gathered twenty articles for this Special Issue that discuss the discovery of novel bioactive NPs with potential for medical purposes. The application of synthetic biology with all its multidisciplinary aspects (bioinformatics, data mining, pathway refactoring, cell factories, DNA editing, and computational chemistry) was preferred to allow the identification of novel drug molecules from microbial strains or bioresources that might escape classical top-down strategies.

The plant kingdom is a significant source of molecules with potential therapeutic benefits for humans; bioactive molecules derived from plants often exhibit clear therapeutic profiles and can be employed as drugs or starting points to derive synthetic drugs [26,27,28,29]. Several recent publications on the biological activity of plant compounds are collected in this Special Issue.

In particular, Sun et al. [30] extracted and purified the lipophilic diterpene Tanshinone IIA (TAN) from *Salvia miltiorrhiz Bunge* and evaluated its role in maintaining chondrocyte viability and promoting cartilage regeneration in osteoarthritis patients. TAN was already recognized in herbal medicine for its anti-inflammatory, antioxidant, and vascular endothelial cell-protective properties. Likewise, Schwarz et al. [31] investigated the mode of action of the steroid sapogenin diosgenin, previously identified in the Chinese plant *Dioscoreae rhizoma,* in dampening the autoimmune inflammatory response in T helper 17 (Th17)-driven pathologies. By combining methodological approaches including gene expression analysis and in silico analyses, the authors revealed diosgenin as an inverse agonist of the key transcription factors leading to Th17 cell differentiation and metabolism. Kim and colleagues [32] demonstrated that the plant-derived ferulic acid acts as a therapeutic agent for wound healing via inhibiting β-catenin in keratinocytes and activating Nrf2 in wound-induced inflammation.

Florets of Safflower (*Carthamus tinctorius*) were identified as a source of polyacetylene glycosides, which are responsible for preventing excessive lipid accumulation in obesity through the inhibition of adipocyte differentiation, reducing the transcription levels of mature adipocyte marker genes (*Adipsin* and *Fabp4*), promoting the expression of lipolytic genes, and downregulating the expression of lipogenic genes [33].

The application of plant-derived compounds (such as polyphenols) in therapies is often hampered by several factors including structural instability; poor bioavailability, gastric solubility, and residence time; and fast metabolization in the liver [34,35,36]. Through the combination of experimental (spectroscopy and calorimetry) and simulation techniques (docking and molecular dynamics simulations) the glycosyl derivate of the flavonoid rutin (quercetin-3-O-rutinose) was found to exhibit comparable potency to the parental molecule rutin and an estimated higher bioavailability. Thus, the results of this work were proposed as the basis for the development of quercetin-like antiviral compounds in coronavirus infection management [37].

Like quercetin, the alkaloid antitumoral camptothecin (CPT) shows weak pharmacokinetic and pharmacodynamics properties [38]. Nanotechnology is perfect for improving CPT bioavailability; thus, the review by Ghanbari-Movahed et al. provided a comprehensive and critical evaluation of the novel, efficient nano-CPT formulations being developed for cancer therapy [38]. By contrast, biotransformation of the antioxidant resveratrol (RSV) by the entomopathogenic fungus *Beauveria bassiana* yielded a safer RSV metabolite, the stilbene glycoside resvebassianol A [39].

As widely reported, NPs can exert multiple biological activities. For example, the anti-inflammatory phenolic compound Apocynin, an inhibitor of NADPH-dependent oxidase (NOX), was suggested to interact with plasmalemmal ionic channels by perturbing ionic currents in excitable cells [40]. However, more evidence is needed to understand the molecular-level nature of interactions affecting neuroendocrine, endocrine, or cardiac function.

Food proteins from animals and plants are widely exploited for cryptic bioactive peptides exhibiting multi-target activities [41,42,43,44,45,46,47,48,49,50]. Gambacorta et al. [51] evaluated the inhibitory activity of the whey-derived bioactive small peptides MHI, IAEK, and IPAVF against the SARS-CoV-2 3C-like protease (3CLpro) for the first time. These peptides were previously obtained by the enzymatic hydrolysis of whey proteins and displayed ACE-inhibitory activity [22]. The authors integrated theoretical and experimental techniques, first performing molecular docking studies to rationally evaluate the putative chance of binding and then in vitro testing for validation. The results confirmed the highest antiviral activity for IPAVF and IAEK, providing new opportunities for the development of dual-target small peptides endowed with antiviral 3CLpro- and ACE-inhibitory activities.

Using a machine learning approach, Casey et al. [52] predicted five novel anti-diabetic peptides (pep_1E99R5, pep_37MB3O, pep_ANUT7B, pep_RTE62G and pep_QT5XGQ) from a set of 10^9^ peptides. Although further work is required to elucidate their bioavailability, mechanism of action, and clinical efficacy, the authors presented pep_1E99R5 as the most active peptide, affecting blood glucose metabolism. Bioactive peptide sequences can also be re-designed to obtain novel drugs in cancer therapy, as reported by [53,54,55]. Thus, an in silico peptide design optimization process was applied to identify active peptides from the C-terminal of azurin, an anticancer bacterial protein produced by *Pseudomonas aeruginosa.* Due to its molecular properties, CT-p19LC was predicted to exhibit the greatest anticancer activity. This was confirmed in experimental trials and, therefore, it was suggested for the development of novel anticancer strategies.

Multidisciplinary approaches effectively reveal the synthesis pathways of NPs [56,57] or discover new molecules that might be used as templates to develop novel biotherapeutics [58,59].

Rugen et al. [60] collected venom from the assassin bug *Rhynocoris iracundus* and investigated its composition and bioactivity in vitro and in vivo to exploit it for biomedical applications. Assassin bug venom induced neurolysis, caused the paralysis and melanization of *Galleria mellonella* larvae and pupae, and exhibited antibacterial activity. The combined proteo-transcriptomic approach could successfully identify molecules responsible for biological effects (redulysins, kininogens, chitinases, hemolysins, and Ptu1 family peptide toxins).

Among 35 phytochemicals, sennoside B from *Cassia angustifolia* was predicted as a TNF-α inhibitor by a competitive binding screening assay coupled with analytical size exclusion chromatography and liquid chromatography–tandem mass spectrometry (LC-MS). Molecular docking was also performed to determine the binding mode of sennoside B to TNF-α, confirming its activity in TNF-α-induced HeLa cell toxicity assays [61]. Similarly, molecular docking revealed that flavonoids (apigenin and luteolin) bound to histone deacetylases (HDACs), with important implications in epigenetic therapy to regulate cellular gene expression [62]. In addition, computational methods were applied to design modified flavonoids endowed with high monoamine oxidase (MAO) B affinity for neurological disorder treatment [63,64], as well as to identify new potential scaffolds against Cyclin-dependent kinase 7 (CDK7) for the development of novel antitumoral strategies [65].

The bioactive compounds found in natural products are also a valuable source of inspiration for new drug synthesis [14,66,67,68], such as the marine-inspired potent kinase inhibitors with antiproliferative activities described by [69]. NPs can also be used to chemically modify the molecular structure of existing drugs to improve their activity or pharmacokinetics properties. For example, Neganova et al. [70] found that the conjugation of sesquiterpene lactones, extracted from *Inula helenium* L. (*Asteraceae*), reduced the side effects of antitumoral canthracycline antibiotics.

In conclusion, the articles published in this Special Issue underline the advances and opportunities in using NPs in drug discovery. Several works show NPs’ key role in a wide array of biological activities, such as maintaining tissue integrity, regulating immune responses, and influencing complex processes in human diseases. Their current limitations and promising strategies to design and identify novel molecules are also discussed.
